# Novel Candidate Genes and a Wide Spectrum of Structural and Point Mutations Responsible for Inherited Retinal Dystrophies Revealed by Exome Sequencing

**DOI:** 10.1371/journal.pone.0168966

**Published:** 2016-12-22

**Authors:** Marta de Castro-Miró, Raul Tonda, Paula Escudero-Ferruz, Rosa Andrés, Andrés Mayor-Lorenzo, Joaquín Castro, Marcela Ciccioli, Daniel A. Hidalgo, Juan José Rodríguez-Ezcurra, Jorge Farrando, Juan J. Pérez-Santonja, Bru Cormand, Gemma Marfany, Roser Gonzàlez-Duarte

**Affiliations:** 1 Departament de Genètica, Microbiologia i Estadística, Facultat de Biologia, Universitat de Barcelona, Barcelona, Spain; 2 Centro de Investigación Biomédica en Red de Enfermedades Raras (CIBERER), Instituto de Salud Carlos III, Barcelona, Spain; 3 Institut de Biomedicina (IBUB), Universitat de Barcelona, Barcelona, Spain; 4 CNAG-CRG, Centre for Genomic Regulation (CRG), Barcelona Institute of Science and Technology (BIST), Barcelona, Spain; 5 Universitat Pompeu Fabra (UPF), Barcelona, Spain; 6 Asociación EsRetina Asturias, Oviedo, Asturias, Spain; 7 Servicio de Oftalmología, Unidad de Retina, Hospital Universitario Central de Asturias, Oviedo, Spain; 8 Stargardt APNES-Retina, Buenos Aires, Argentina; 9 Hospital Interzonal General de Agudos Eva Perón, Buenos Aires, Argentina; 10 Barraquer – Centro de Oftalmología Barcelona, Barcelona, Spain; 11 Institut Oftalmològic Quirón Barcelona, Barcelona, Spain; 12 Department of Ophthalmology, Alicante University General Hospital, Alicante Institute for Health and Biomedical Research (ISABIAL-FISABIO Foundation), Alicante, Spain; King Faisal Specialist Hospital and Research Center, SAUDI ARABIA

## Abstract

**Background:**

NGS-based genetic diagnosis has completely revolutionized the human genetics field. In this study, we have aimed to identify new genes and mutations by Whole Exome Sequencing (WES) responsible for inherited retinal dystrophies (IRD).

**Methods:**

A cohort of 33 pedigrees affected with a variety of retinal disorders was analysed by WES. Initial prioritization analysis included around 300 IRD-associated genes. In non-diagnosed families a search for pathogenic mutations in novel genes was undertaken.

**Results:**

Genetic diagnosis was attained in 18 families. Moreover, a plausible candidate is proposed for 10 more cases. Two thirds of the mutations were novel, including 4 chromosomal rearrangements, which expand the IRD allelic heterogeneity and highlight the contribution of private mutations. Our results prompted clinical re-evaluation of some patients resulting in assignment to a syndromic instead of non-syndromic IRD. Notably, WES unveiled four new candidates for non-syndromic IRD: *SEMA6B*, *CEP78*, *CEP250*, *SCLT1*, the two latter previously associated to syndromic disorders. We provide functional data supporting that missense mutations in *CEP250* alter cilia formation.

**Conclusion:**

The diagnostic efficiency of WES, and strictly following the ACMG/AMP criteria is 55% in reported causative genes or functionally supported new candidates, plus 30% families in which likely pathogenic or VGUS/VUS variants were identified in plausible candidates. Our results highlight the clinical utility of WES for molecular diagnosis of IRD, provide a wider spectrum of mutations and concomitant genetic variants, and challenge our view on syndromic vs non-syndromic, and causative vs modifier genes.

## Introduction

Massive sequencing, particularly Whole Exome Sequencing (WES), has completely revolutionized genetic diagnosis of highly heterogeneous monogenic disorders. In the field of inherited retinal disorders (IRD), more than 20 novel genes, mostly identified by WES, have been reported since the beginning of 2014 (an average of one novel gene per month). This success relies on the power of primary sequence DNA data at a genomic scale, the increasing number of suitable up-to-date databases of SNP allelic frequencies in different populations, the relative simplicity of standardized WES protocols and the availability of powerful and increasingly refined bioinformatics tools [1–4].

The molecular diagnosis yield of WES is highly empowered by complementary genetic data (e.g. homozygosity mapping and linkage analysis), which greatly favours the identification of the causative gene in recessive cases. In contrast, finding the pathogenic mutation in dominant cases amidst the high number of heterozygous variants identified by WES is far from trivial, and often requires cosegregation analysis in large pedigrees, which are not always available neither necessarily conclusive [5–8].

Molecular diagnosis of IRD is one of the main aims of our research. Currently, aside WES, other massive sequencing-based approaches have been also implemented in IRD genetic diagnosis laboratories, such as targeted-sequencing of a limited set of causative/candidate genes [9–12]. These approaches have proved useful to study large cohorts in order to identify reported or novel mutations in known genes, but they may fall short when the pathogenic mutation maps in an unreported candidate. Increasing the number of analysed genes greatly redounds in the final diagnostic efficiency, broadens the spectrum of the cellular pathways underlying the retinal pathological state, provides invaluable insights into phenotype-modifier genes and opens new venues for therapy [13,14].

We have used WES to diagnose a cohort of families affected of a wide spectrum of IRD, including syndromic and non-syndromic cases, recessive and dominant families as well as sporadic cases. Initially, a single individual from each family was assessed by WES, followed by Sanger sequencing as well as cosegregation analysis in available members. A total of 18 out of 33 cases were finally diagnosed, 17 showing causative mutations in already reported genes. In other 10 families, plausible candidates complying with some of the ACMG/AMP criteria were identified. Since most of the mutations are novel, including gross deletions and duplications, our results illustrate the high allelic heterogeneity of IRD and highlight the contribution of private mutations. Most important, we propose four new IRD candidates based on the WES data, genetic cosegregation, *in silico* and functional analyses, thus increasing the genetic factors and cellular pathways underlying neurodegeneration.

## Materials and Methods

### Subjects

A total of 33 families from Argentina, Saudi Arabia and Spain with patients diagnosed with IRD were recruited from reference ophthalmological institutions or patient’s associations. Peripheral blood DNA from patients and available relatives was obtained using the QIAamp DNA Blood Maxi Kit (Qiagen, Hilden, Germany). Written informed consent from all patients and relatives was obtained following the tenets of the Declaration of Helsinki. Procedures for patient recruitment and sample collection were previously approved by the Bioethics Committee of the University of Barcelona (Barcelona, Spain).

### Library preparation and sequencing

Exome sequencing was performed at the Centro Nacional de Análisis Genómico (CNAG, Barcelona, Spain). Paired-end multiplex libraries were prepared with Illumina TruSeq DNA Sample Prep kit (Illumina, San Diego, California, USA) and enriched with the Agilent SureSelect Human AllExon v5 (Agilent, Santa Clara, California, USA). Libraries were loaded onto Illumina flowcells for cluster generation prior to producing 100 base read pairs on a HiSeq2000 instrument. Base calling, quality control and data processing was performed with the Illumina RTA sequence analysis pipeline as previously described[15]. Detected mutations were verified by Sanger sequencing. Coverage of the target region and the subset of retinitis related genes were assessed with DepthOfCoverage from GATK. Subsets of variants falling in the capture region were obtained using Bedtools[16] and common unix commands.

### Prioritization of genetic variants

First, variants that altered the coding region of IRD genes were retrieved when the allelic frequency in public databases (ExAC[17]) was lower than or equal to 1%. Therefore, only infrequent null, missense, frameshift and splicing mutations were considered. The second filter was that the number of identified mutations complied with the expected Mendelian pattern of inheritance. Third, the missense variants were considered pathogenic when at least two of the in silico prediction algorithms (MutationTaster[18] SIFT[19], PolyPhen2[20] and CADD[21]) gave a positive score. Selected variants were validated by Sanger sequencing, and confirmed by cosegregation whenever possible. Pathogenicity was considered according to the ACMG/AMP standards and guidelines, which take into account.

### Detection of chromosomal rearrangements

Coverage analysis was visualized using IGV[22]. For pedigrees 10NCE and 68ORG, cosegregation of SNPs mapping at the expected deleted region was analysed by PCR amplification and sequencing as aforementioned. Multiplex Ligation-dependent Probe Amplification (MLPA) analysis of *PRPF31* was carried on 23 individuals of family E4, using the MLPA Retinitis Pigmentosa kit SALSA-P235-B2 (MRC Holland, Amsterdam, The Netherlands) with probes for every *PRPF31* exon. MLPA products were separated by capillary electrophoresis and analysed using the software Coffalyser v8 (MRC Holland) to evaluate CNVs, considering a deletion when the probe ratio between sample and control was under 0.7, and duplication when it was over 1.3. The breakpoint of the *CRX* deletion in family 10NCE was mapped using common SNPs between *CRX* and *SULT2A1* for PCR-amplification and Sanger-sequencing to identify heterozygosity in affected probands (inferring non-deletion) or homozygosity for different alleles in mother and child (inferring deletion). Adjacent regions were aligned using Pairwise Sequence Alignment[23]. In family E4, we considered and finally demonstrated a tandem duplication of *PRPF31* exons 2 to 5, by genomic DNA amplification using specific PCR primers and subsequent sequencing.

### Immunodetection of CEP250 in the mouse retina

Adult C57BL/6J mice (IMSR_JAX:000664) were sacrificed in accordance with National and European regulations. For immunocytochemistry, eyes from adult C57BL/6J mice (IMSR_JAX:000664) were removed and fixed in 0.1 M phosphate buffer, containing 4% formaldehyde for 4 hr at RT. Tissues were cryopreserved overnight in 30% sucrose, embedded in OCT (Sakura Finetek, Leiden, The Netherlands) and snap-frozen. Cryosections (12 μm) were incubated with 0.3% Triton X-100 and 2% sheep serum in PBS at 4°C with the corresponding primary antibodies: anti-CEP250 1:100 (AB_2076918, Proteintech, Chicago, USA), anti-rhodopsin 1D4 1:200 (AB_304874, Abcam, Cambridge, UK) and anti-acetylated α-tubulin 1:3000 (AB_477585, Sigma, St. Louis, Missouri, USA). After incubation with Alexa Fluor 488 and 568 secondary antibodies and DAPI (5 μg/ml) (Sigma) for 2 h at room temperature, slides were mounted with Mowiol. Confocal images were obtained using a TCS SP2 confocal microscope (Leica Microsystems, 63× objective, 1.4 NA).

### Expression of wt and mutant CEP250 constructs in ARPE19 cells

The mammalian expression vector (pCMV6 backbone) containing the human Wt-CEP250 full-length coding region was obtained from OriGene. Site-directed mutagenesis was then performed on the Wt-CEP250 sequence to generate the A609V mutant (Mt-CEP250). The IT6 epitope (Antibody BCN, Cerdanyola del Vallès, Spain) sequence was fused at the C-terminus of each coding region. All constructs were sequenced to confirm integrity. Human ARPE-19 cells (1.5x10^5^/well) were plated (40% confluence) onto poly-L-lysine coated coverslips in 1:1 Ham’s F10 and DMEM containing 10% fetal bovine serum and 1% penicillin-streptomycin. After 24 h, cells were transfected with 0.8 μg/well of either wild-type or mutant CEP250-IT6 expression constructs using Lipofectamine 2000 (Invitrogen, Heidelberg, Germany). On reaching confluence, cells were deprived of serum for 48 h and then fixed with paraformaldehyde for 30 min at room temperature. Coverslips were washed 3 times with PBS and processed for immunofluorescence staining. Labelling was performed with anti-IT6 antibody 1:100 (kindly provided by Antibody BCN), anti-acetylated α-tubulin 1:3000 (AB_477585, Sigma) and DAPI nuclear counterstaining. Confocal images were acquired sequentially using a TCS SP5 confocal microscope (Leica Microsystems, 100× objective, 1.4 NA). Cilia length was measured using Fiji implementation of Image J[24] and statistical analysis was carried on with GraphPad Prism.

## Results

### WES results and genetic analysis of pedigrees

A total of 33 families affected by syndromic and non-syndromic IRD were recruited and analysed by WES for genetic diagnosis after clinical evaluation. Fourteen patients were simplex cases with no reported genetic precedent. Four families showed a dominant Mendelian pattern of inheritance, whereas fourteen were compatible with recessive inheritance. We were able to identify the genetic cause of the disease in 18 families (out of 33). Moreover, credible candidates are proposed for 10 more cases. Therefore, the strict global diagnostic efficiency was 55% to which an additional 30%, based on the identification of plausibly pathogenic variants, could be added.

For all tested samples (46 individuals belonging to the 33 pedigrees), median coverage in the whole capture region was 54.90 ± 11.26 (mean 60x) with more than 96% of the targeted bases covered by 10 or more reads. Equivalent coverage results were observed when looking only at the IRD genes (based on RetNet[25]). We designed systematic filtering steps for the selection and prioritization of variants, within the framework proposed by ACMG/AMP standards and guidelines for evaluating pathogenicity. Only 5 cases remained undiagnosed because no clear disease-causing candidate was(were) identified.

Overall, the results of the WES analysis globally revealed a mean of 43597 variants in the whole exome per individual, of which 958 were located in known IRD genes. In several families, patients were carriers of additional IRD-pathogenic recessive mutations in heterozygosis, which could certainly add to the phenotypic severity and variability.

### Detailed genotype analysis

The results of WES are shown in Table 1. Concerning the syndromic cases, only one pedigree (79ORG), affected by Usher, had a correct clinical diagnosis. The two other pedigrees, previously diagnosed as non-syndromic IRD cases, turned out to be syndromic after WES-mediated identification of the causative genes and mutations. A young patient (simplex case, 64ORG) carried two mutations in the *PHYH* gene, encoding a peroxisomal enzyme associated to the Refsum syndrome. Retinitis Pigmentosa (RP) is an early-onset symptom of this syndrome followed later on by other pathological traits (e.g. deafness, skin alterations, peripheral neuropathy and ataxia). One allele was a frameshift mutation leading to protein truncation, and the second allele (P223R) caused a damaging missense substitution in a conserved residue embedded in a highly evolutionarily conserved PhyH domain (S1 Fig). Clinical and metabolic re-evaluation of the pre-symptomatic patient (phytanic acid levels in serum measured by gas chromatography were 32 times the normal threshold values) totally confirmed the Refsum syndrome diagnosis, which had been previously unnoticed, and allowed to implement a preventive treatment for late-onset Refsum disorder traits, by decreasing the ingestion of phytanic acid. According to the clinician, the patient has since greatly improved in some phenotypic traits, mainly reducing the ichthyosis bursts and the anosmia. Similarly, two novel mutations in the *C21orf2* gene, a frameshift and a damaging missense substitution in a highly conserved position within the leucine-rich repeats (S1 Fig), re-classified a non-syndromic cone-rod dystrophy to the recently reported spondylometaphyseal dysplasia syndrome (SMD), where RP is associated to severe thoracic abnormalities. Patients in this family (A10) showed both RP and skeletal dysplasia, initially considered as unlinked traits. Again, our results prompted clinical re-evaluation, and supported *C21orf2* association to this syndrome[26].

**Table 1:** Disease-causing mutations and presumptive pathogenic variants identified by WES in our cohort of IRD patients.

Family	Disease	Gene	Mutation	Zygo-sity	Cosegre- gation available	Computational prediction							Allele frequency	Reported	ACMG classifi-cation
						Polyphen2		SIFT		MutationTaster		CADD			
						Effect	Score	Effect	Score	Effect	Score		ExAC		
**A) Syndromic Causative Genes**															
**A10**	ar axial SMD	*C21orf2*	c.286G>A p.E96K	Het	Yes	Probably D.	1	Tolerated	NA	Disease C.	1	36	Not present	Novel	Likely P.
			c.631_632del p.R211Hfs*46	Het		-	-	-	-	Disease C.	1	-	Not present	Novel	Pathog.
**64ORG**	ar Refsum	*PHYH*	c.668C>G p.P223R	Het	Yes	Probably D.	1	Tolerated	0,06	Disease C.	1	22.7	Not present	Novel	Likely P.
			c.683dupG p.V229Sfs*2	Het		-	-	-	-	Disease C.	1	-	2/121412*	[27]	Pathog.
**79ORG**	arUSH	*USH2A*	c.2299delG p.E767Sfs*21	Het	Yes	-	-	-	-	-	-	-	96/121284	[28]	Pathog.
			c.9119G>A p.W3040*	Het		-	-	Tolerated	1	Disease C.	1	52	Not present	[29]	Pathog.
**B) Non-syndromic Causative Genes**															
77ORG	arCD	*ABCA4*	c.3386G>T p.R1129L	Het	Yes	Probably D.	0,961	Damaging	0	Disease C.	1	28.01	30/121388*	[30]	Likely P.
			c.4539+2064C>T	Het		-	-	-	-	Polymorphism	0	-	Not Covered	[31]	VUS
A18	arRP	*BBS2*	c.334T>C p.F112L	Hom	Yes	Probably D.	1	Damaging	0,01	Disease C.	1	28.8	1/121022*	Novel	VUS
**55ORG**	arCRD	*C2orf71*	c.1067_1068del p.N356Rfs*101	Hom	No	-	-	-	-	Disease C.	1	-	Not present	Novel	Pathog.
65ORG	arLCA	*CEP290*	c.148C>T p.H50Y	Het	No	Possibly D.	0,952	Damaging	0,05	Disease C.	1	25	Not present	Novel	VUS
			c.1322T>A p.L441*	Het		-	-	Tolerated	1	Disease C.	1	41	Not present	Novel	Pathog.
67ORG	arRP	*CERKL*	c.613+5_613+8del	Hom	Yes	-	-	-	-	Disease C.	1	-	Not present	Novel	VUS
75ORG	arAchr	*CNGA3*	c.1768G>A p.E590K	Hom	Yes	Probably D.	0,954	Damaging	0,05	Disease C.	1	22.8	1/121004*	[32]	VUS
**80ORG**	arRP	*CNGB1*	c.2762_2765delACGA p,Y921Cfs*15	Hom	Yes	-	-	-	-	-	-	-	Not present	Novel	Pathog.
**71ORG**	arAchr	*CNGB3*	c.1148delC p.T383Ifs*13	Hom	Yes	-	-	-	-	Disease C.	1	-	224/120952^#^	[33]	Pathog.
**2ORG**	arRP	*CRB1*	c.2688T>A p.C896*	Het	No	-	-	Tolerated	1	Disease C.	1	23.2	2/121386*	[34]	Pathog.
			c.2842T>C p.C948R	Het		Probably D.	0,996	Damaging	0	Disease C.	1	15.2	Not present	Novel	Likely P.
**10NCE**	adRP	*CRX*	Deletion exons 3–4	Het	Yes	-	-	-	-	-	-	-	-	Novel	Pathog.
**68ORG**	arRP	*EYS*	c.2380C>T p.R794*	Het	Yes	-	-	-	-	Disease C.	1	37	1/19764*	Novel	Pathog.
			Deletion 10 initial exons	Het		-	-	-	-	-	-	-	-	Novel	Pathog.
**58ORG**	arLCA	*GUCY2D*	c.914delA p.H305Pfs*90	Hom	Yes	-	-	-	-	Disease C.	1	-	Not present	Novel	Pathog.
**69ORG**	arGF	*NRL*	c.339C>G p.Y113*	Hom	Yes	-	-	-	-	Disease C.	1	37	Not present	Novel	Pathog.
**81ORG**	adOA	*OPA1*	c.800_801delAA p.K267Rfs*4	Het	Yes	-	-	-	-	-	-	-	1/120600*	Novel	Pathog.
**E4**	adRP	*PRPF31*	Partial deletion & duplication	Het	Yes^1^	-	-	-	-	-	-	-	-	Novel	Pathog.
**76ORG**	XlRP	*RPGR*	c.762_777delinsCA p.T255Rfs*23	Hem	Yes	-	-	-	-	-	-	-	Not present	Novel	Pathog.
**82ORG**	adRP	*UNC119*	c.7delG p.V3*	Het	No	-	-	-	-	-	-	-	Not present	Novel	Likely P.
**51ORG**	arRP	*USH2A*	c.1724G>A p.C575Y	Het	Yes	Probably D.	1	Damaging	0	Disease C.	1	16.57	1/121370*	[35]	Likely P.
			c.2276G>T p.C759F	Het		Probably D.	0,999	Damaging	0	Disease C.	1	23.1	95/121178*	[36]	Likely P.
**73ORG**	arRP	*USH2A*	c.2276G>T p.C759F	Het	Yes	Probably D.	0,999	Damaging	0	Disease C.	1	23.1	95/121178*	[36]	Likely P.
			c.13010C>T p.T4337M	Het		Probably D.	0,986	Damaging	0	Disease C.	1	24.8	Not present	[37]	Likely P.
**C) Uncertain cases**															
22ORG	RP	*CNGB3*	c.1672G>T p.G558C	Het	Yes^2^	Probably D.	1	Damaging	0	Disease C.	1	26.8	4/119080*	[33]	VUS
		*CRB1*	c.1702C>T p.H568Y	Het		Probably D.	0,997	Tolerated	1	Disease C.	1	16.43	Not present	[38]	VUS
		*ROM1*	c.668G>A p.R223Q	Het		Probably D.	0,999	Tolerated	0,09	Disease C.	1	36	17/121408*	Novel^3^	VUS
39ORG	RP	*PDE6B*	c.928-9_940dup p.Y314Cfs*50	Het	Yes^2^	-	-	-	-	-	-	-	Not present	[39]	Pathog.
		*USH2A*	c.1246G>T p.A416S	Het		Probably D.	0,998	Tolerated	0,1	Disease C.	1	24.5	1/117386*	Novel	VUS
**D) New Candidates / New phenotypes for reported syndromic genes**															
**A3**	arRP	*CEP250*	c.1826C>T p.A609V	Hom	Yes	Probably D.	NA	Damaging	NA	Neutral	NA	20.6	33/121314*	Novel	Likely P.
56ORG	arRP	*CEP78*	c.1056delT p.T353Lfs*5	Hom	Yes	-	-	-	-	Disease C.	1	-	Not present	Novel	VGUS
62ORG	arRP	*SCLT1*	c.778-2A>T	Het	Yes	-	-	-	-	Disease C.	1	19.45	Not present	Novel	Pathog.
			c.827G>A p.R276H	Het		Probably D.	0,998	-	-	Disease C.	1	20.6	3/121324*	Novel	VUS
66ORG	arCRD	*SEMA6B*	c.493G>A p.G165R	Hom	Yes	Possibly D.	0,814	Damaging	0,03	Disease C.	0,995	14.13	1/118664*	Novel	VGUS

Phenotype, inheritance model, causative gene and mutation, zygosity, co-segregation analysis, *in silico* pathogenicity predictions, allelic frequency in control population and ACMG classification of variants are indicated per each family. 1: Two different mutations segregate in the pedigree; 2: Assuming incompleate penetrance of the mutation; 3 Mutation p.R223W has been previously reported in a dominant patient (Xu et al 2014);

*All individuals carried the variant in heterozygosity;

^#^ One individual carries the variant in homozygous state.

GF: Goldmann-Favre; SMD: Spondylometaphyseal Dysplasia; D: Damaging; Disease C: Disease Causing; NA:no available. PATH: pathogenic; VUS: Variant of uncertain significance.; VGUS: Clearly disruptive variant in a gene of uncertain significance

Families with secure genetic diagnosis are highlighted in bold.

With respect non-syndromic cases, WES identified the causative mutations in IRD reported genes in 21 cases/families (Table 1). Mild mutations in syndromic genes have already been reported as causative for non-syndromic phenotypes. This is also the case in our cohort for mutations in *BBS2* (pedigree A18), *CEP290* (pedigree 65ORG) and *USH2A* (51ORG and 73ORG). The *BBS2* and *CEP290* mutations are novel, contrary to those of *USH2A*. Mutations in *CEP290* are associated to severe ciliopathies, except for a prevalent intronic mutation that affects the splicing pattern and decreases the pool of full-length molecules. Remarkably, the two mutations here identified in heterozygosis expand the panoply of non-syndromic *CEP290* mutations. Since one allele is null (Table 1), the missense substitution H50Y, residing in a non-strictly conserved peptide stretch within the homo/heterodimerization domain, should be relatively mild (S1 Fig).

Although most of the identified alleles were novel mutations in genes already assigned to a defined clinical entity, clinical assignment was sometimes reconsidered after genetic diagnosis, e.g. patients previously assigned as arLCA were re-classified as affected from autosomal recessive achromatopsia, after identification of mutations in *CNGA3* and *CNGB3*.

**Fig 1:**
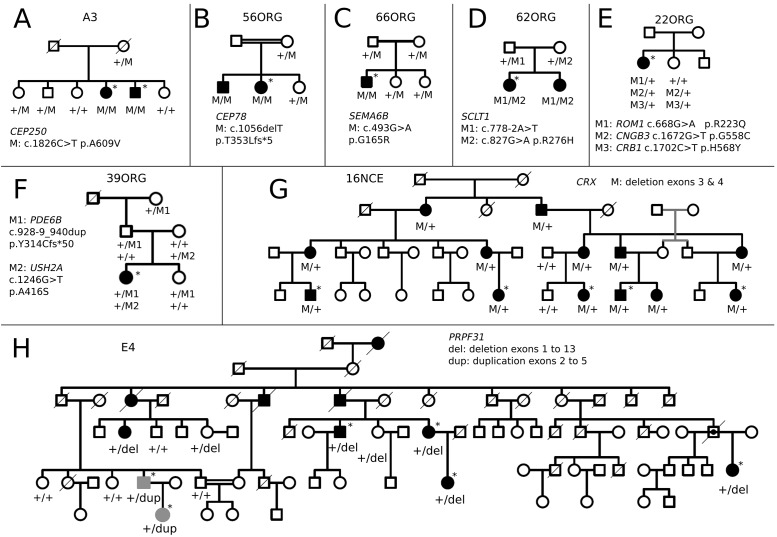
Segregation of mutations in selected pedigrees. Pedigrees bearing new IRD candidates and chromosomal rearrangements are shown. Pedigrees where mutations in several genes co-segregate with the disease are also depicted. Alleles and carrier status are indicated below each analysed individual. Grey symbols (in H) shown patients bearing a different chromosomal rearrangement. The rest of the pedigrees are available as S3 Fig.

Simplex cases are usually assumed to be recessive. However, in our cohort, four simplex patients (nearly 20%) were either hemizygous or heterozygous for genes associated to X-linked or dominant mutations. For instance, a male patient showed a pathogenic indel in exon 7 of the *RPGR* gene. Besides, we identified a heterozygous deletion in exon 1 of *UNC119* in the 82ORG case, further supporting this gene as candidate for dominant IRD. Remarkably, reported dominant mutations in *CNGB3* and *PDE6B* were respectively identified in families 22ORG and 39ORG (Fig 1). However, this mutational assignment is controversial according to our results, since cosegregation analysis showed other carrier members that were unaffected, even after clinical re-evaluation. Notably, the affected individuals also carried heterozygous missense mutations in other reported genes, which were absent in the non-affected carriers of *CNGB3* and *PDE6B* dominant mutations (Fig 1, Table 1), suggesting that incomplete penetrance could be due to independent segregation of two different mutations, only detectable after WES.

**Fig 2:**
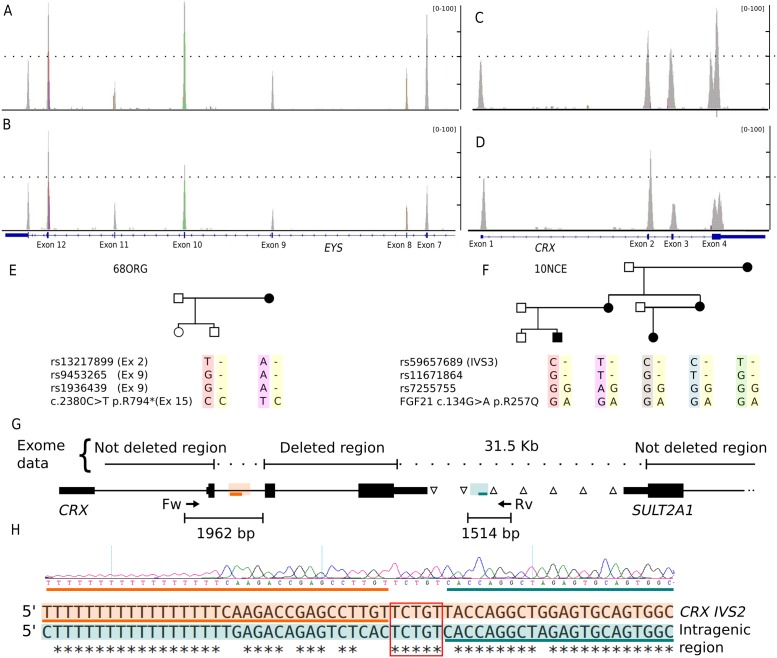
Identification of *EYS* and *CRX* deletions. A-F) Two different gross heterozygous deletions in genes *EYS* and *CRX* were respectively identified as the causative mutation in families 68ORG and 10NCE. The probands (B and D) showed a reduction in the coverage of some exons compared to the respective controls (A and C). The segregation of SNPs located in the expected deleted region showing that mother and child were homozygous for different alleles is indicated below. (E and F). G) Chromosomal deletion in family 10NCE is defined by genotyping common SNPs between *CRX* and *SULT2A1* genes in the affected probands. Heterozygous SNPs are indicated by △, whereas SNPs where mother and child were homozygous for different alleles are indicated by ∇. Adjacent breakpoint regions with high sequence similarity are boxed in orange and green and preserved sequences in the rearranged allele are indicated with orange and green lines. H) Sequence chromatogram of the rearranged allele is shown below. Alignment of the highly similar sequences of *CRX* intron 2 (CRX IVS2) and the intragenic region involved in the rearrangement is also indicated. Again, orange and green lines are the adjacent sequences to the breakpoint, which is indicated by a red square.

**Fig 3:**
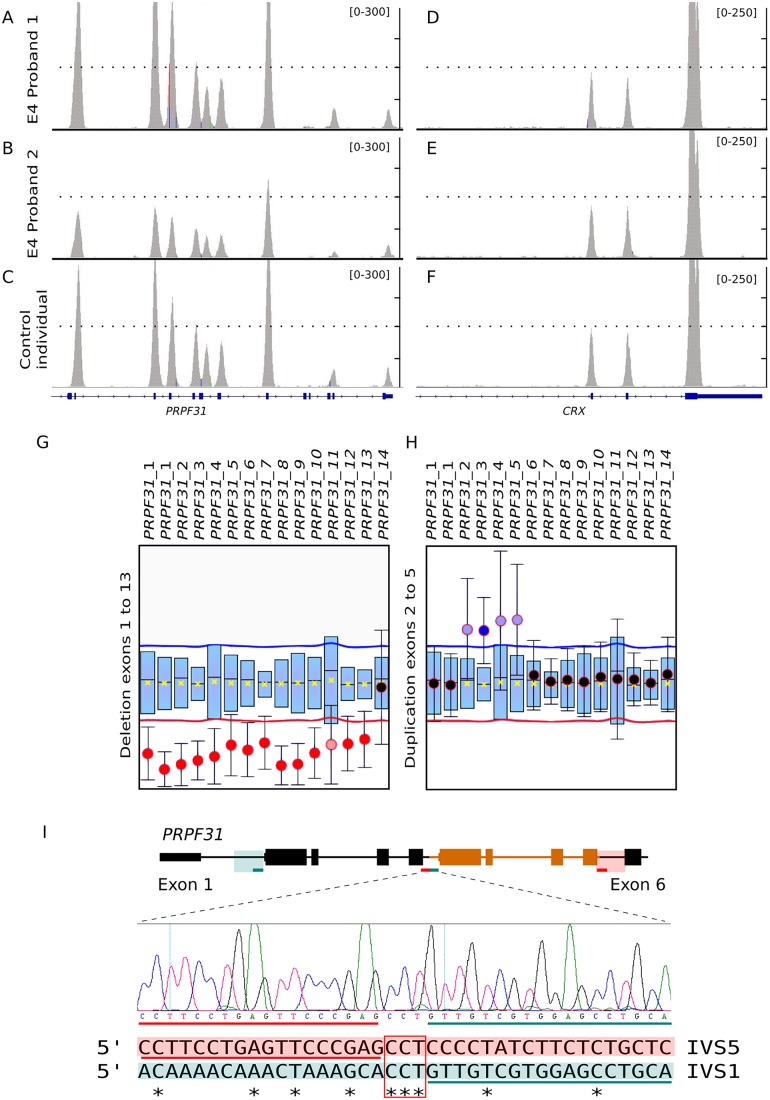
Identification of independent *PRPF31* deletion and duplication segregating in pedigree E4. Exome data indicated significant coverage differences of *PRPF31* exons in the E4 family, pointing to chromosomal rearrangements. Some patients (A) showed higher coverage in exons 2–5 compared to a control sample (C) whereas patients from another family branch showed a significant decrease of exons 1–13 (B). *CRX*, located a few Mb away from *PRPF31* gene, was used as a control gene (D-F). MLPA analysis confirmed a nearly full deletion of *PRPF31* (exons 1 to 13) in some patients of the family (G) and an internal duplication involving exons 2 to 5 in other affected members (H) (shown in grey in Fig 1). I) Chromosomal region of *PRPF31* involved in the duplication, where the duplicated exons are coloured in orange. Green and red lines below indicate the extent of the duplication. Chromatogram of the rearranged allele is shown below. Alignment of the flanking sequences (boxed in orange and green) involved in the rearrangement shows no clear homology. Orange and green lines are the adjacent sequences to the breakpoint, which is indicated by a red square.

Large deletions and chromosomal rearrangements were identified by WES in three families. In the arRP 68ORG family, a single nonsense pathogenic allele in the *EYS* gene was first identified. A careful coverage analysis along all the *EYS* exons unveiled a novel deletion encompassing the initial 10 exons as a second allele. This deletion was confirmed by homozygous SNP haplotype in the affected mother and carrier son (Fig 2). In two very large dominant pedigrees, WES did not render any plausible candidate. In family 10NCE, a very rare and mild polymorphism mapping in a gene adjacent to *CRX*, cosegregated with the disease. A careful coverage analysis of *CRX* in the patients identified a heterozygous deletion encompassing exons 3 and 4 (Fig 2), which was finely mapped by genomic DNA PCR and Sanger sequencing. The flanking regions shared high sequence similarity probably account for illegitimate homologous recombination. This result prompted us to re-evaluate the WES coverage data in all dominant unsolved pedigrees. Remarkably, the large E4 pedigree showed an unusual chromosomal rearrangement (Fig 3), since one side of the family showed increased exon coverage in the *PRPF31* gene, whereas the other side of the family showed a decrease. To confirm and map the exons involved, a complete *PRPF31* MLPA analysis was performed and two rearranged pathogenic alleles segregating in the same family were identified: an internal duplication affecting exons 2 to 5 (inclusive) and a gross deletion involving exon 1 to 13 of *PRFP31*. Since WES coverage in the upstream genes was also decreased, this deletion could include nearby genes, at least *TFPT NDUFA3*, and a part of *OSCAR*.

### New candidates

When no pathogenic variants were identified in IRD genes, we expanded the mutational search to all the WES data. Following the ACMG/AMP guidelines, four novel recessive candidates were proposed based on the mutational effect at the molecular level, the predicted *in silico* pathogenicity and the assigned gene function. These four candidates are *SEMA6B* and three members of the centrosomal/ciliary protein family *CEP78*, *CEP250*, *SCLT1*. In all these cases, the clinical assessment of the patients was only RP (S2 Fig).

*SEMA6B*, mutated in homozygosis in a simplex case, is highly expressed in the development of the murine retina and belongs to a family of transmembrane proteins with multiple roles in signalling and axon guidance[40,41]. The G165R missense variant is located within the semaphorin domain, close to the plexin binding sites. This substitution changes the size and charge of a conserved amino acid in vertebrates (S1 Fig), and is predicted *in silico* as pathogenic (Table 1). Moreover, the mutation strictly cosegregates with the disease in the family (Fig 1).

*CEP78* encodes a centrosomal protein of unknown function. Our rationale behind proposing this gene as probably causative of arRP is: i) affected individuals are homozygous for the mutation, which causes a frameshift that generates a truncated protein, ii) mutations on several centrosomal protein genes cause IRD, and finally, iii) cosegregation with the disease in the family (Fig 1).

*SCLT1* was also proposed to be causative of early-onset RP since: i) this gene causes oro-facial-digital syndrome type IX, a very rare and severe ciliopathy with congenital eye defects; ii) patients are heterozygotes for one missense and one splicing altering mutations; and indeed iii) the variants cosegregate with the disease in the family (Fig 1). The missense mutation, R276H, alters a relevant residue in the Smc multidomain (chromosome segregation ATPase domain) shared with many other proteins, many of them centrosomal, involved in cell cycle control[42]. Therefore, we here describe a new phenotype for a previously reported ciliopathy gene.

**Fig 4:**
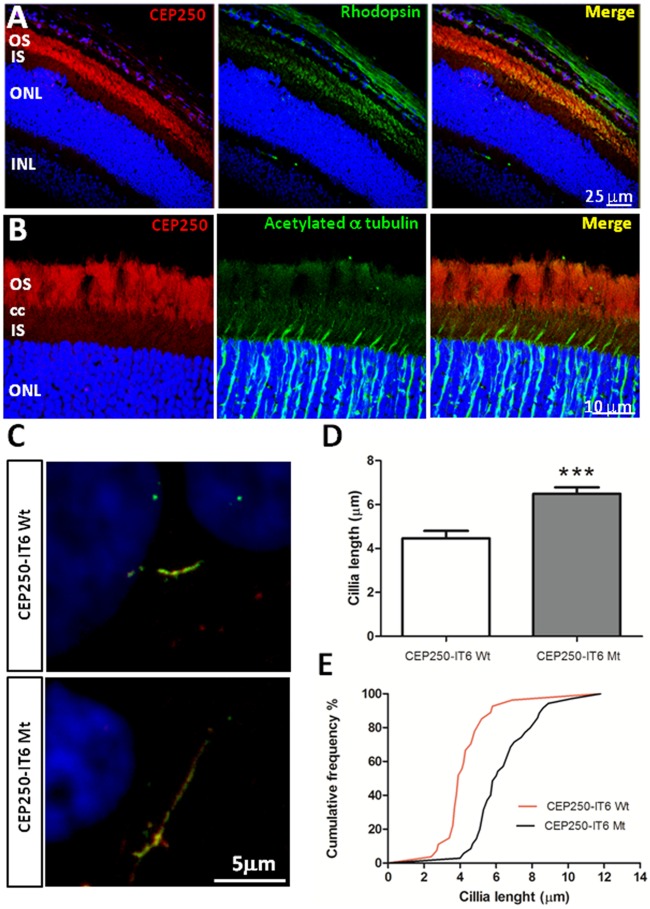
Immunodetection of endogenous CEP250 in mouse retinal cryosections. Immunostaining of CEP250 with rod photoreceptor marker rhodopsin (A) and acetylated α-tubulin (B). CEP250 stained mainly the outer segment of photoreceptors (CEP250 is in red, Rhodopsin and acetylated-α-tubulin in green, nuclear counterstaining by DAPI in blue). **Cells expressing A609V CEP250-IT6 show longer cilia**. (C) Wild-type (Wt) and mutant (Mt) CEP250-IT6 (green) co-localize with acetylated α-tubulin (red) to primary cilia in serum-starved ARPE-19 cells. Immunolabelling of CEP250 and acetylated α-tubulin show longer cilia in cells transfected with the mutant A609V CEP250-IT6 compared to Wt-CEP250-IT6. (D) Cilia length quantification in Wt- and Mt- CEP250-IT6 transfected cells. Graph shows that cilia from cells expressing mutant CEP250 were one third longer than cilia from cells expressing Wt-CEP250 (n>30). Mean and error are shown. *** indicates high statistical significance by the t-Student test, p<0.001. (E) Distribution of cilium length represented as a cumulative frequency chart of the percentage of total cilia. OS—photoreceptor outer segments; CC—connecting cilium; IS—photoreceptor inner segments; ONL—outer nuclear layer; INL—inner nuclear layer; GCL—ganglion cell layer.

Concerning *CEP250*, previously associated to atypical Usher syndrome, the homozygous missense A609V is a seemingly mild amino acid substitution embedded in a long evolutionarily conserved stretch of the Smc domain. Its presumed pathogenicity was based on *in silico* prediction algorithms and cosegregation with the disease. However, since this substitution only affected size but not the charge or other chemical properties of the amino acid, we decided to test CEP250 expression in the retina, and assay the phenotypic effects of the mutation. Endogenous CEP250 in mouse retinal cryosections (P60) was mostly detected in the outer photoreceptor segment (Fig 4A), supporting its localization in the photoreceptor axoneme (Fig 4B). Other centrosomal proteins that cause IRD, such as OFD1, share a similar localization pattern in the retina[43]. Furthermore, localization of the wild-type and A609V CEP250 proteins was studied in transfected human ARPE19 cells where cilia formation was promoted after serum-starvation. To avoid steric hindrance we used a new epitope specifically designed for innocuous protein tagging (IT6, Antibody BCN). The endogenous CEP250 expression was not silenced, but the use of the IT6 antibody allowed us to identify transfected cells. Remarkably, cells expressing the A609V CEP250 (Mt-CEP250) protein consistently showed elongated primary cilia compared to Wt-CEP250 transfected cells (Fig 4C). Cilium length was measured and quantified. On average, Mt-CEP250 cilia were one third longer than the wild-type counterparts, with high statistical significance (Fig 4D and 4E). Overall, our results support involvement of CEP250 in retinal ciliogenesis, and highlight this gene as a new IRD candidate.

In many patients, besides the causative variants, additional rare variants, predicted as damaging by at least two *in silico* algorithms, were identified in known IRD genes. These variants were mostly unreported in the Human Gene Mutation Database[44]. We did not consider them as causative on the grounds of heterozygosis in recessive probands, lack of cosegregation in dominant cases, relative frequency of homozygotes in the reported normal population (ExAC), and because of the identification of two mutations in a more plausible pathogenic candidate in the proband. We deem these variants (S1 Table) are likely modifiers of the phenotypic traits of the patients.

## Discussion

The WES analysis (mean coverage around 60x) of our cohort of 33 unrelated families has identified the causative gene in 18 cases and proposed a plausible candidate in 10 additional cases. Only five cases (15%) remained unsolved. Overall, we have identified 21 unreported mutations in known IRD genes and additionally proposed four novel candidates, *CEP250*, *CEP78*, *SEMA6B* and *SCLT1*. Functional support for the pathogenicity of the homozygous A609V missense mutation in *CEP250* is also provided. Our prioritized analysis of WES is very efficient and comparable to other NGS-based methods, such as NGS-customized targeted sequencing [45]. However, syndromic genes which would be rarely included in gene panels can be easily analyzed. Besides, when no mutations in the prioritized genes are found, the search for pathogenic variants in new candidates can be performed using the same data by changing the search parameters. This new search can: 1) highlight presymptomatic patients of severe late-onset disorders, 2) uncover new phenotype-genotype associations in milder alleles of syndromic genes, 3) propose new IRD candidates.

Concerning clinical re-evaluation, pathogenic variants in *PHYH* (a syndromic gene usually absent in customized targeted panels) were identified in patient 64ORG, which questioned the initial diagnosis of RP, informing the clinician and the patient of the disease before other Refsum syndrome traits appeared and most importantly, leading to a preventive treatment. In family A10, the mutations in *C21orf2* also changed the initial CRD diagnosis to axial spondylometaphyseal dysplasia, associating RP to previously unrelated skeletal traits. Besides, WES also uncovered new mutations in very low prevalence genes, not usually included in targeted panels, e.g. *UNC119*, in our cohort [11,46,47].

The ever increasing number of novel mutations—even in previously reported genes—highlighted by massive sequencing is changing the scenario of established genotype-phenotype correlations and patterns of mendelian inheritance[48] and calls for an in-depth re-evaluation of our genetic knowledge[49]. Double heterozygosity of private mutations may be more frequent than expected in recessive cases of “rare-but-not-so-rare” diseases (prevalence 1:3000), barring the cases of highly consanguineous populations, where homozygosity mapping has been the basic genetic tool. On the other hand and concerning dominant cases, two previously reported dominant mutations affected a single member of the family in contrast to a larger number of asymptomatic carriers. These contradictory results might be explained by either misassignment of the pathogenicity of the genetic variant or incomplete gene screening in past reports.

We have identified several pathogenic duplications and deletions of large chromosomal regions in already reported genes in two dominant (affecting *CRX* and *PRPF31*) and in one recessive pedigree (altering *EYS*). Remarkably, in the E4 dominant pedigree (Fig 1), both a deletion and a duplication in *PRPF31* were identified in two branches of the same family. *PRPF31* pathogenic alleles due to internal deletions/duplications are not infrequent and amount up to 20% [50]. The remarkable finding is that two of such highly disruptive chromosomal rearrangements segregated in two branches of the same family. The rearranged genomic regions differed in size, involved different exons, and did not share any apparent flanking sequence, which would suggest independent genetic events (Fig 3). These structural mutations are still difficult to identify in heterozygosis unless specific dosage analysis tests or careful evaluation of coverage are performed. However, our results strongly support the relevant contribution of structural variation to disease (50% of our dominant cases). We deem it likely that the list of pathogenic alleles involving chromosomal rearrangements will increase in the following years.

Four new candidates for non-syndromic IRD were also highlighted by WES. Remarkably, three of them are related to cilia and ciliogenesis (*SCLT1*, *CEP250* and *CEP78*). Severe gene mutations involved in ciliogenesis are either lethal or cause severe syndromic ciliopathies, whereas milder alleles show a more restricted phenotype and affect only particular ciliated organs (retina, cochlea, renal tubules…)[51], e.g. *CEP290* cause Leber Congenital Amaurosis[52], and *OFD1* is responsible for severe X-linked RP[53] as well as oro-facial-digital syndrome I, characterized by craniofacial, oral and skeletal abnormalities[54] or other syndromic diseases[55]. We have identified a mild missense substitution and a mutation in the consensus acceptor splicing site (intron 10) of *SCLT1*, another member of the oro-facial-digital syndrome gene family[56]. These previous reports highlight *SCLT1* as a new arRP candidate, and illustrate the tenuous line between genes involved in syndromic and non-syndromic disorders.

Our WES results further support the involvement of two CEP proteins in IRD. A homozygous frameshift mutation in *CEP78* in two siblings of a consanguineous family pointed directly to this gene as a plausible new candidate for arRP. Concerning *CEP250* involvement in retinal neurodegeneration, previous reports described a close interaction with *NEK2* (a centrosomal RP gene)[57,58], and it was also suggested as a modifier gene in an atypical Usher syndrome family where nonsense mutations in *CEP250* in homozygosis and in *C2orf71* in heterozygosis showed an additive effect[59]. Our results support that a homozygous missense mutation in *CEP250* may cause arRP by altering the structure and length of cilia. Of note, in this family another arRP mutation segregated in heterozygosis, the reported C948Y *CRB1* mutation (frequent in the Spanish population[60,61]), which could also contribute to the severity of the phenotype.

WES data allows the identification of the causative mutations plus incidental findings in other relevant retinal genes. When focusing solely on IRD genes, many patients are heterozygous carriers of both, known pathogenic recessive alleles (average of 0.3 mutations per patient), and rare predicted pathogenic variants (average of 5.25 variants per patient). Similar values of heterozygous carriers of pathogenic variants in IRD genes have been identified in control individuals (1:4) after NGS-analysis[62]. Most of these recessive alleles correspond to prevalent mutations in the geographical population of origin and thus, heterozygous carriers should be expected among controls. Therefore, databases of human genetic variation of healthy individuals used as controls for new pathogenic variants may in fact contain true mutations. Indeed, caution should be recommended in genetic diagnosis to avoid misinterpretation of these findings. On the other hand, when drawing genotype-phenotype correlations in relation with the clinical prognosis, these additional recessive genetic variants might act as hypomorphic alleles that induce or enhance the phenotypic effects of the causative mutations, accounting for the phenotypic differences within carriers of the same mutations. For instance, the 22ORG pedigree showed heterozygous variants in *CRB1* and *ROM1* besides the reported dominant mutation in *CNGB3*. The patient bears the *CNGB3* mutation together with the *ROM1* mutation, whereas the non-penetrant sibling only carries the *CNGB3* mutation (Fig 1). Similarly, the patient in family 39ORG carried a heterozygous mutation in *USH2A* additionally to the previously reported dominant mutation in *PDE6B*, in contrast to several non-penetrant members of the family who only carried the *PDE6B* pathogenic variant. Therefore, the definition of modifier or susceptibility genes may be widened to include heterozygous mutations in already known causative genes, which probably underlie reduced penetrance and variable expressivity, and calls for reconsideration of the concepts of causative, susceptibility and modifier genes.

Overall, our work supports WES as a highly informative and effective approach for patient molecular diagnosis in retinal dystrophies provided there is qualified genetic counselling. Besides, our data contributes to the databases that aim to address personalized genotype-phenotype correlations. Genomic data from carefully clinically diagnosed patients are required to build a comprehensive human genomic landscape and elucidate how the interactions and complex tradeoffs between mutations, additional genetic variants—and environmental factors—tip the scales towards pathogenicity or resilience to disease.

## Supporting Information

S1 Fig Conservation of missense mutations. Clustalw alignment of protein sequences of different species: Human, Macaque, Rabbit, Mouse, Opossum, Chicken, *X*. *tropicalis*, Stickleback, Zebrafish, *D*. *melanogaster* and *C*. *elegans* (when available) are shown. The position of the mutated amino acid is highlighted with a red box.
(PDF)

S2 Fig Fundus eye photographs. Right and left fundus eye images are shown from affected members of families A3 (A and B), 62ORG (C and D) and 56ORG (E and F) with mutations in *CEP250*, *SCLT1* and *CEP78*, respectively. All these patients have been diagnosed with Retinitis Pigmentosa.
(PDF)

S3 Fig Cosegregation analysis of the mutations identified by WES in our cohort (complementary to Fig 1). Probands sequenced by WES are indicated with an asterisk (*).
(PDF)

S1 Table Summary of exome data per sample. For each sample, total coverage as well as data restricted to IRD genes is shown. For IRD genes, the number of known heterozygous recessive mutations (excluding the causative mutations in the family) and the number of unreported heterozygous variants predicted as pathogenic at least by two different algorithms, are indicated. C10 and C30 are the percentage of bases with a minimum coverage of 10x and 30x, respectively.
(XLS)

S2 Table Criteria used to classify pathogenic variants according ACMG guidelines. (XLS)
