# Supplementary material for: Novel Candidate Genes and a Wide Spectrum of Structural and Point Mutations Responsible for Inherited Retinal Dystrophies Revealed by Exome Sequencing
Source: PLoS One. 2016 Dec 22;11(12):e0168966. doi: 10.1371/journal.pone.0168966 (PMC5179108; doi:10.1371/journal.pone.0168966)

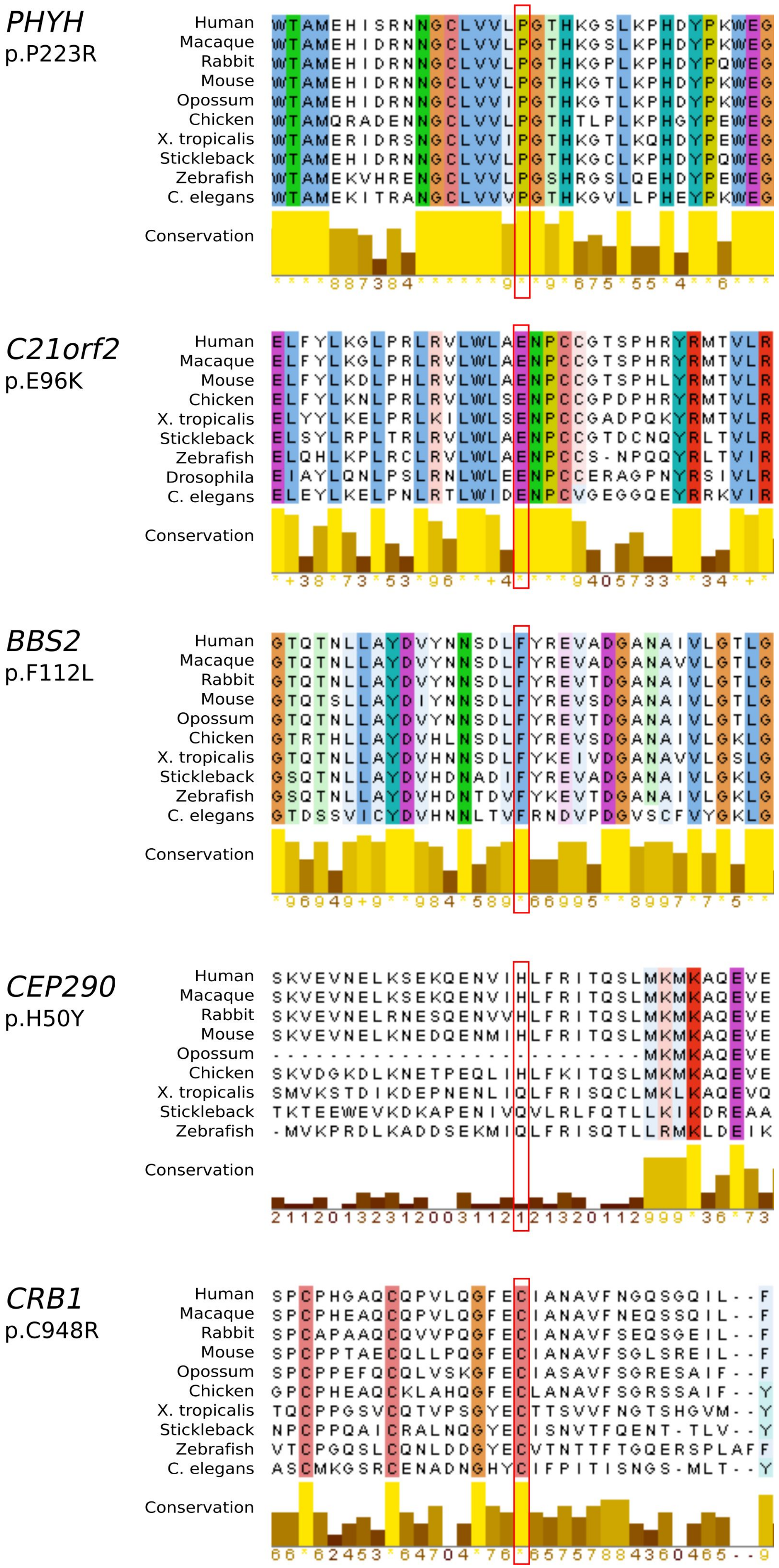
PHYH
p.P223R
Human
Macaque
Rabbit
Mouse
Opossum
Chicken
X. tropicalis
Stickleback
Zebrafish
C. elegans
Conservation
C21orf2
p.E96K
Human
Macaque
Mouse
Chicken
X. tropicalis
Stickleback
Zebrafish
Drosophila
C. elegans
Conservation
BBS2
p.F112L
Human
Macaque
Rabbit
Mouse
Opossum
Chicken
X. tropicalis
Stickleback
Zebrafish
C. elegans
Conservation
CEP290
p.H50Y
Human
Macaque
Rabbit
Mouse
Opossum
Chicken
X. tropicalis
Stickleback
Zebrafish
Conservation
CRB1
p.C948R
Human
Macaque
Rabbit
Mouse
Opossum
Chicken
X. tropicalis
Stickleback
Zebrafish
C. elegans
Conservation

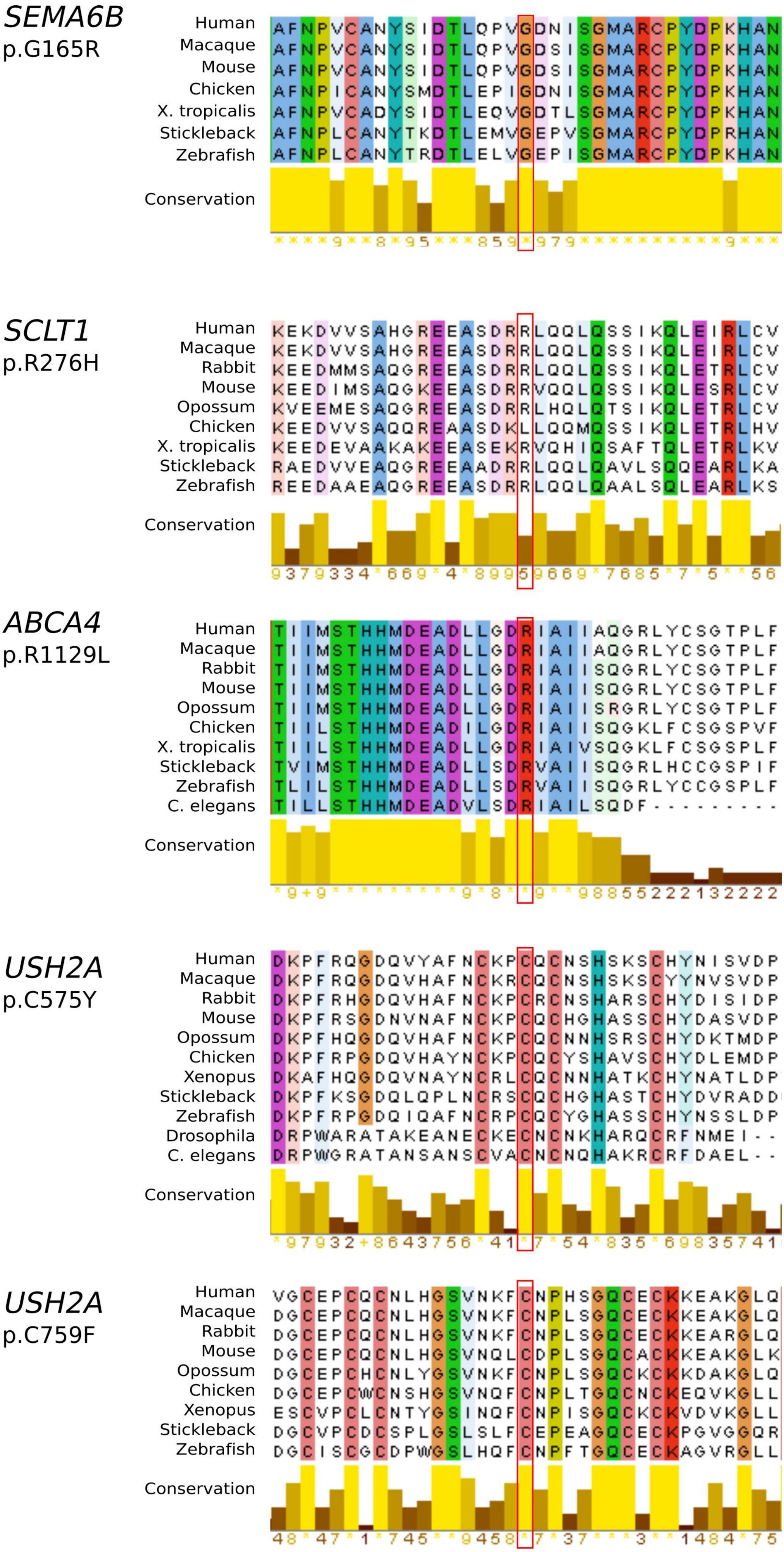
SEMA6B
p.G165R
Human
Macaque
Mouse
Chicken
X. tropicalis
Stickleback
Zebrafish
Conservation
SCLT1
p.R276H
Human
Macaque
Rabbit
Mouse
Opossum
Chicken
X. tropicalis
Stickleback
Zebrafish
Conservation
ABCA4
p.R1129L
Human
Macaque
Rabbit
Mouse
Opossum
Chicken
X. tropicalis
Stickleback
Zebrafish
C. elegans
Conservation
USH2A
p.C575Y
Human
Macaque
Rabbit
Mouse
Opossum
Chicken
Xenopus
Stickleback
Zebrafish
Drosophila
C. elegans
Conservation
USH2A
p.C759F
Human
Macaque
Rabbit
Mouse
Opossum
Chicken
Xenopus
Stickleback
Zebrafish
Conservation

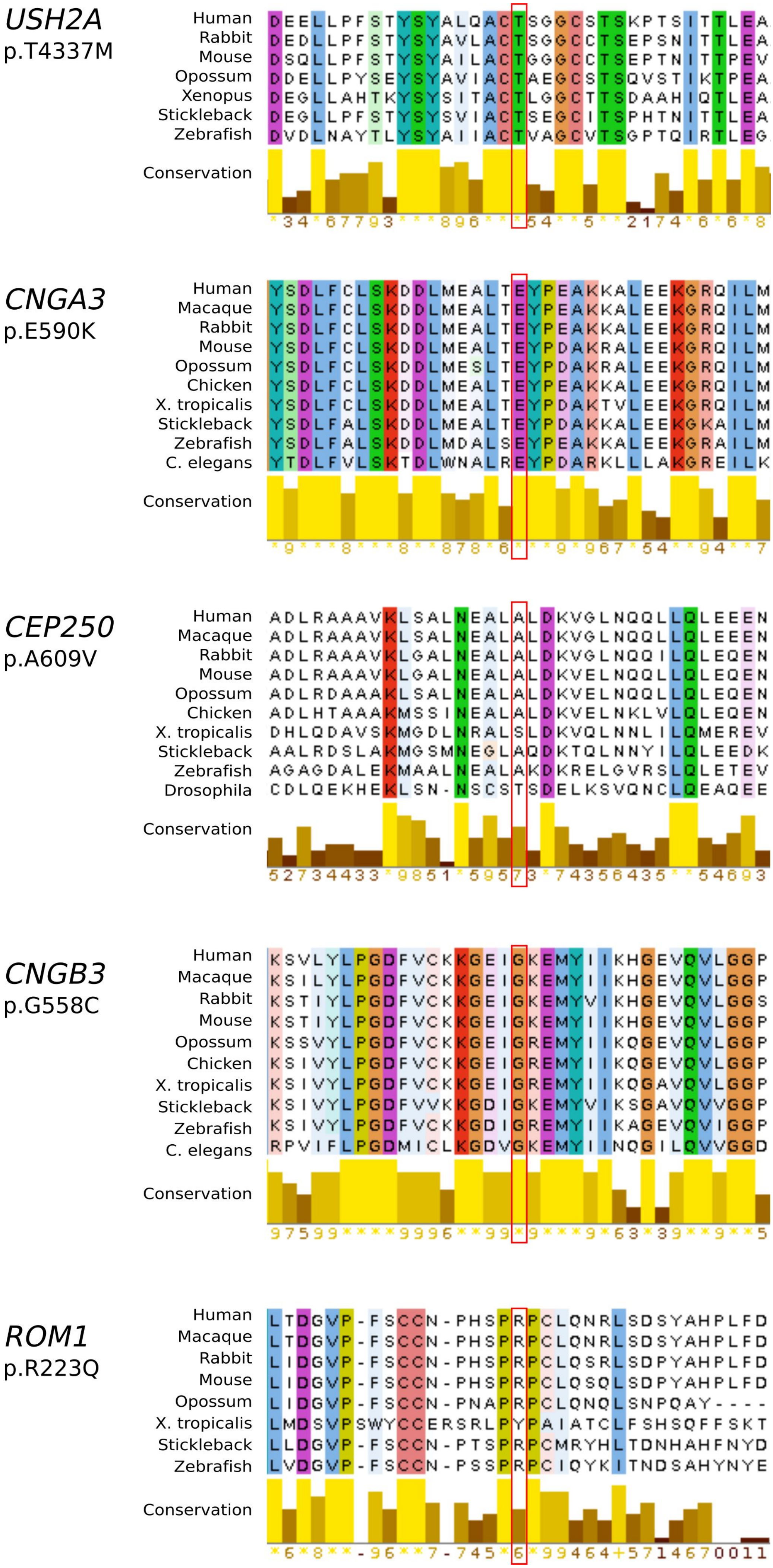
USH2A
p.T4337M
Human
Rabbit
Mouse
Opossum
Xenopus
Stickleback
Zebrafish
Conservation
CNGA3
p.E590K
Human
Macaque
Rabbit
Mouse
Opossum
Chicken
X. tropicalis
Stickleback
Zebrafish
C. elegans
Conservation
CEP250
p.A609V
Human
Macaque
Rabbit
Mouse
Opossum
Chicken
X. tropicalis
Stickleback
Zebrafish
Drosophila
Conservation
CNGB3
p.G558C
Human
Macaque
Rabbit
Mouse
Opossum
Chicken
X. tropicalis
Stickleback
Zebrafish
C. elegans
Conservation
ROM1
p.R223Q
Human
Macaque
Rabbit
Mouse
Opossum
X. tropicalis
Stickleback
Zebrafish
Conservation

USH2A
p.A416S

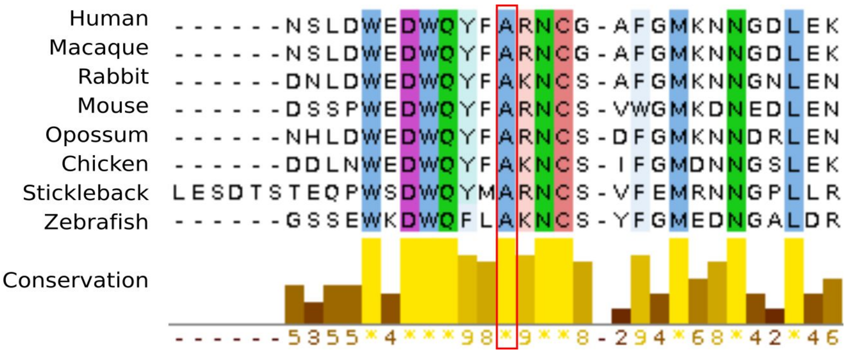

Supplement: S1 Fig — Clustalw alignment of protein sequences of different species: Human, Macaque, Rabbit, Mouse, Opossum, Chicken, X. tropicalis, Stickleback, Zebrafish, D. melanogaster and C. elegans (when available) are shown. The position of the mutated amino acid is highlighted with a red box. (PDF) [file pone.0168966.s001.pdf]
