# Supplementary figures and images for: Novel Candidate Genes and a Wide Spectrum of Structural and Point Mutations Responsible for Inherited Retinal Dystrophies Revealed by Exome Sequencing
Source: PLoS One. 2016 Dec 22;11(12):e0168966. doi: 10.1371/journal.pone.0168966 (PMC5179108; doi:10.1371/journal.pone.0168966)

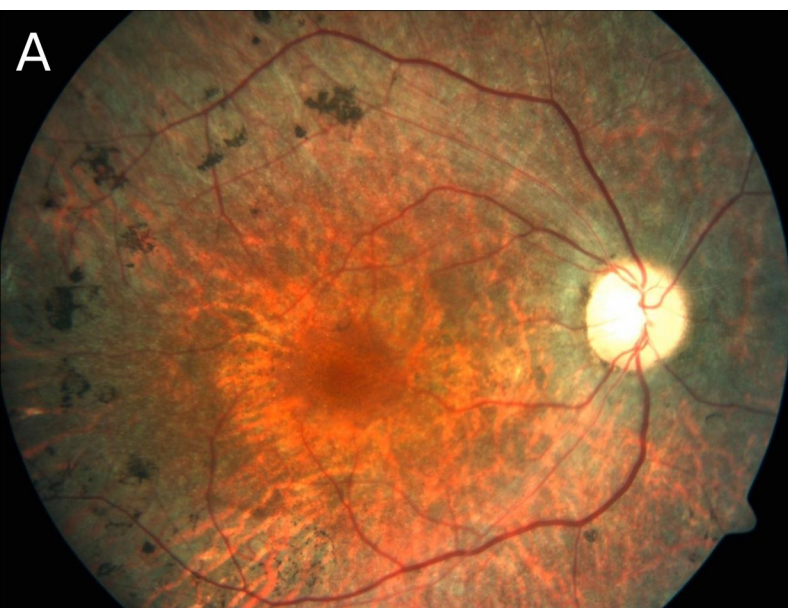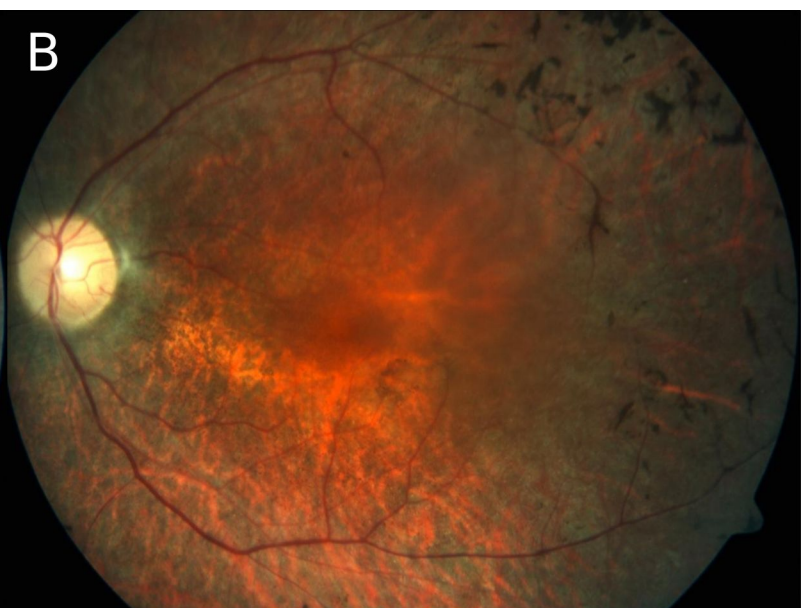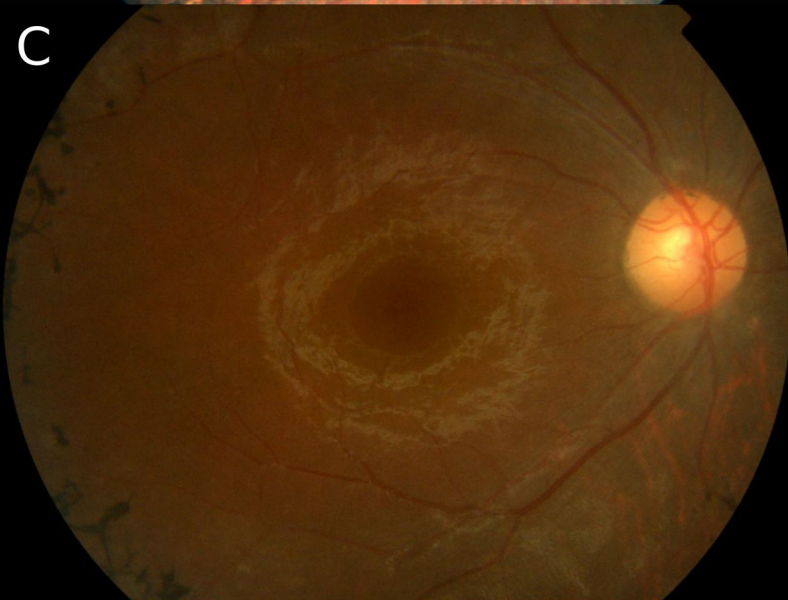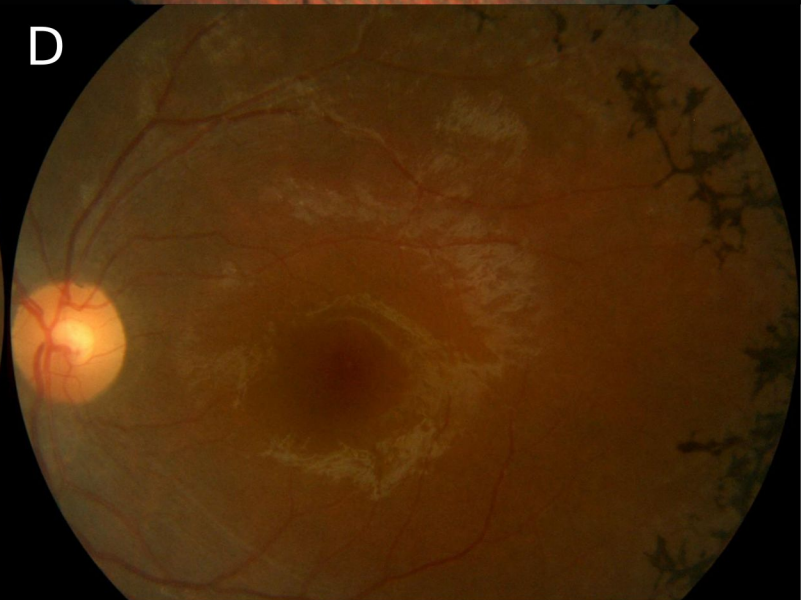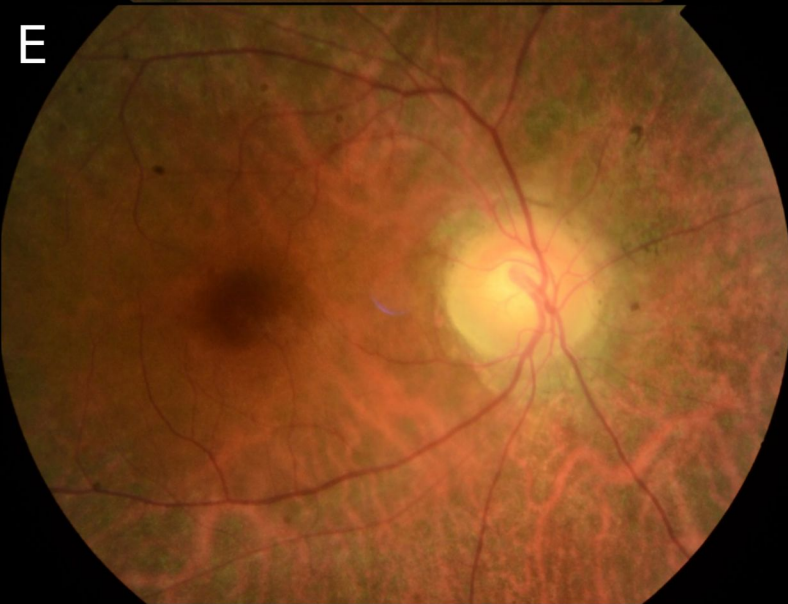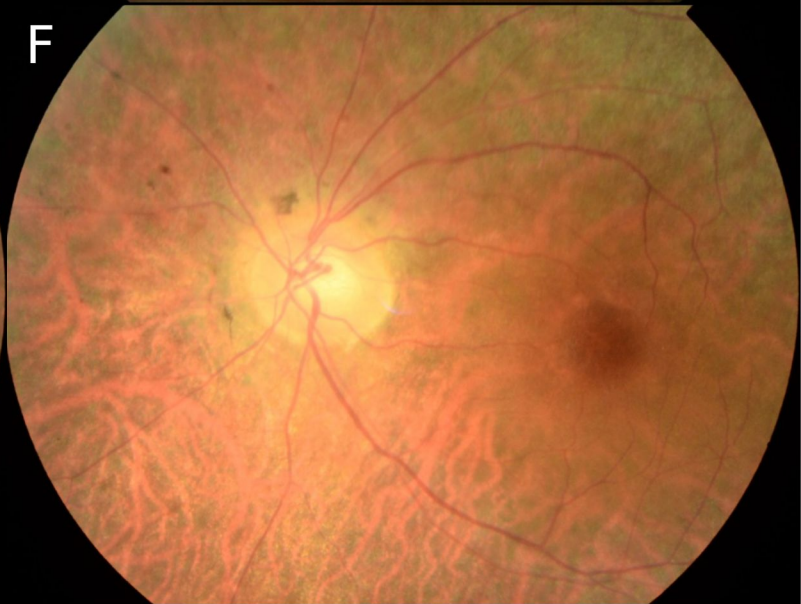

Supplement: S2 Fig — Right and left fundus eye images are shown from affected members of families A3 (A and B), 62ORG (C and D) and 56ORG (E and F) with mutations in CEP250, SCLT1 and CEP78, respectively. All these patients have been diagnosed with Retinitis Pigmentosa. (PDF) [file pone.0168966.s002.pdf]
