# Supplementary material for: Novel Candidate Genes and a Wide Spectrum of Structural and Point Mutations Responsible for Inherited Retinal Dystrophies Revealed by Exome Sequencing
Source: PLoS One. 2016 Dec 22;11(12):e0168966. doi: 10.1371/journal.pone.0168966 (PMC5179108; doi:10.1371/journal.pone.0168966)
Supplement: S3 Fig — Probands sequenced by WES are indicated with an asterisk (*). (PDF) [file pone.0168966.s003.pdf]

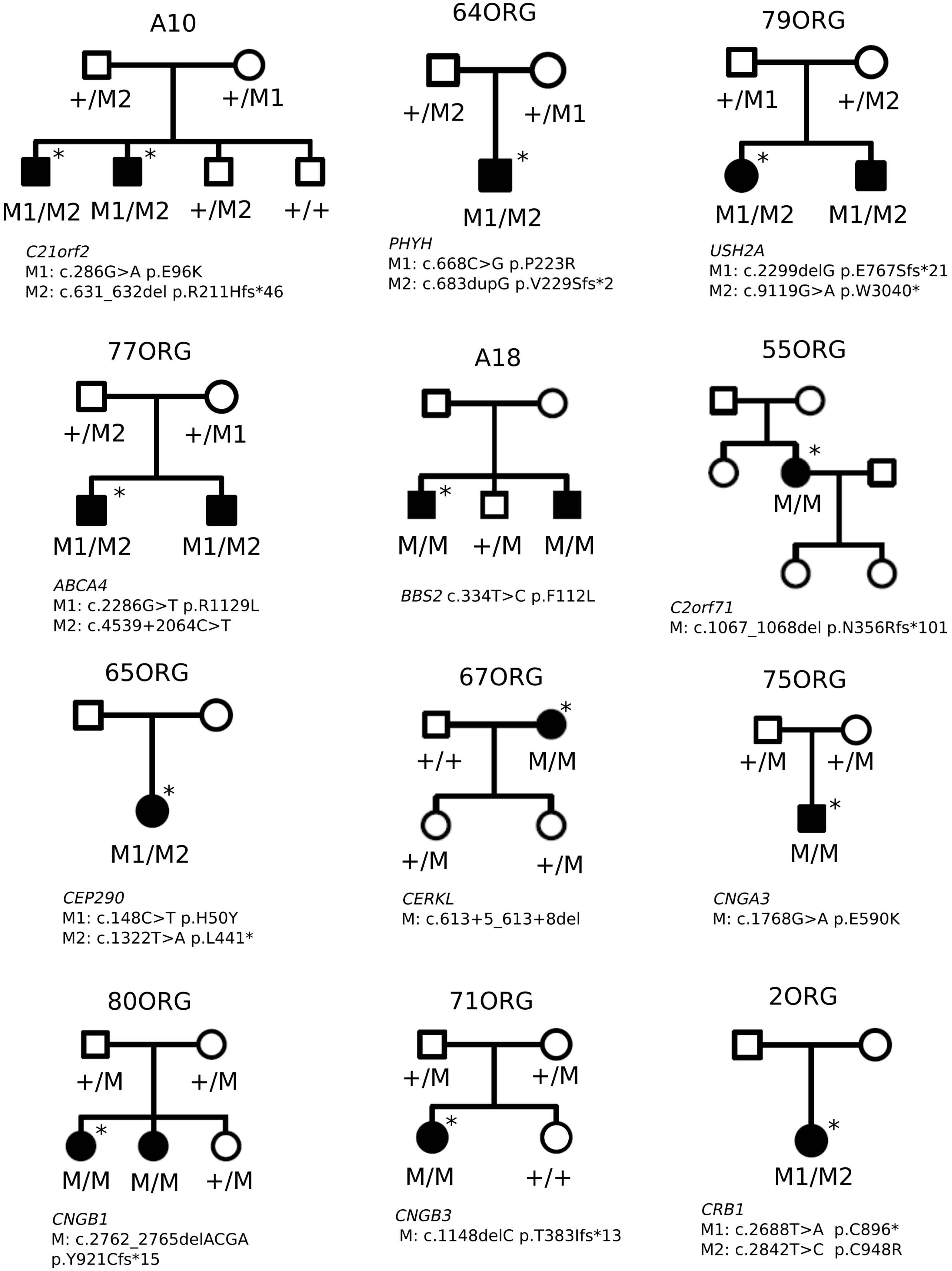
A10
+/M2
+/M1
*
*
M1/M2
M1/M2
+/M2
+/+
C21orf2
M1: c.286G>A p.E96K
M2: c.631_632del p.R211Hfs*46
64ORG
+/M2
+/M1
*
M1/M2
PHYH
M1: c.668C>G p.P223R
M2: c.683dupG p.V229Sfs*2
79ORG
+/M1
+/M2
*
M1/M2
M1/M2
USH2A
M1: c.2299delG p.E767Sfs*21
M2: c.9119G>A p.W3040*
77ORG
+/M2
+/M1
*
M1/M2
M1/M2
ABCA4
M1: c.2286G>T p.R1129L
M2: c.4539+2064C>T
A18
*
M/M
+/M
M/M
BBS2 c.334T>C p.F112L
55ORG
*
M/M
C2orf71
M: c.1067_1068del p.N356Rfs*101
65ORG
*
M1/M2
CEP290
M1: c.148C>T p.H50Y
M2: c.1322T>A p.L441*
67ORG
*
+/+
M/M
+/M
+/M
CERKL
M: c.613+5_613+8del
75ORG
+/M
+/M
*
M/M
CNGA3
M: c.1768G>A p.E590K
80ORG
+/M
+/M
*
M/M
M/M
+/M
CNGB1
M: c.2762_2765delACGA
p.Y921Cfs*15
71ORG
+/M
+/M
*
M/M
+/+
CNGB3
M: c.1148delC p.T383Ifs*13
2ORG
*
M1/M2
CRB1
M1: c.2688T>A p.C896*
M2: c.2842T>C p.C948R

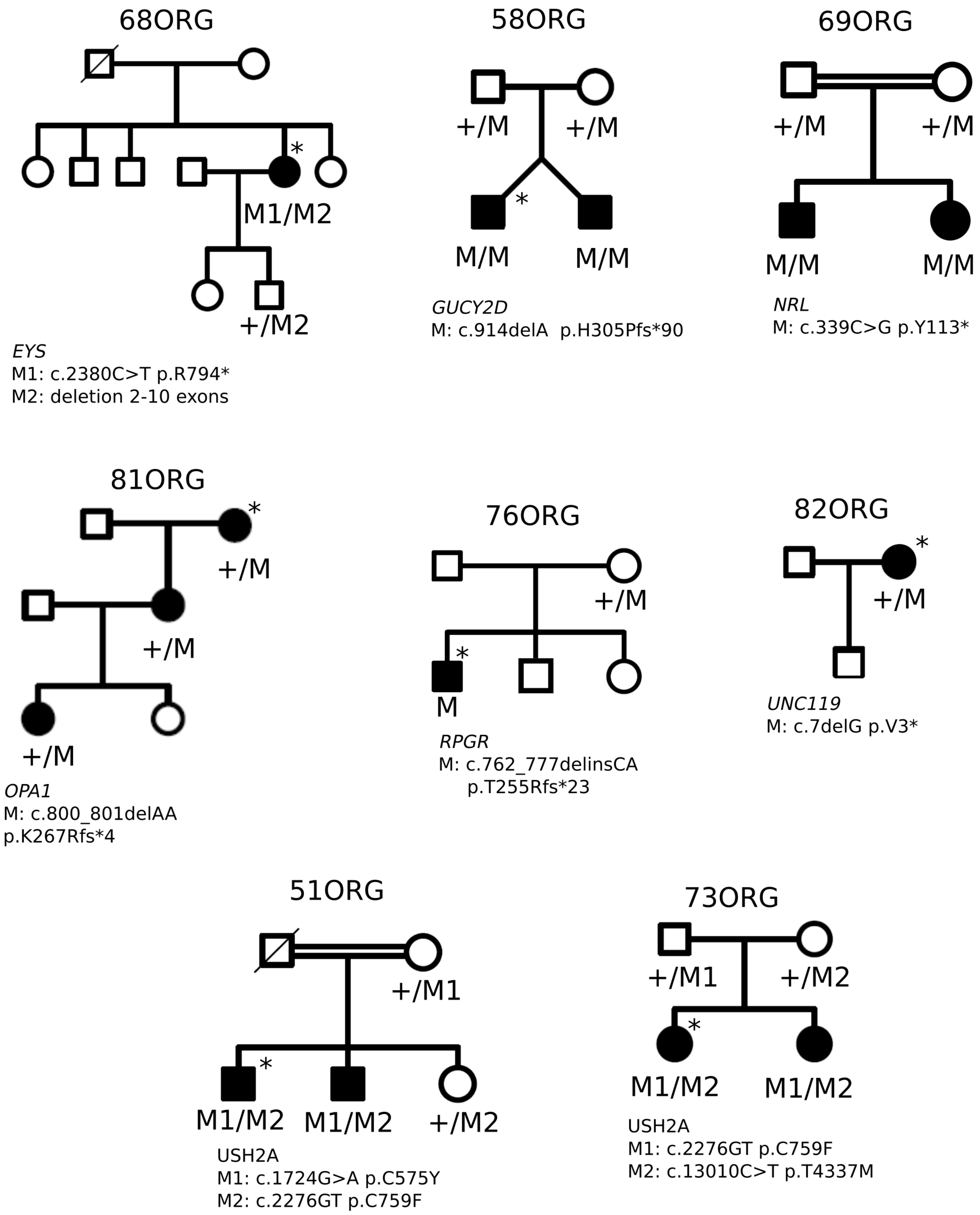
68ORG
*
M1/M2
+/M2
EYS
M1: c.2380C>T p.R794*
M2: deletion 2-10 exons
58ORG
+/M
+/M
*
M/M
M/M
GUCY2D
M: c.914delA p.H305Pfs*90
69ORG
+/M
+/M
M/M
M/M
NRL
M: c.339C>G p.Y113*
81ORG
*
+/M
+/M
+/M
OPA1
M: c.800_801delAA
p.K267Rfs*4
76ORG
+/M
*
M
RPGR
M: c.762_777delinsCA
p.T255Rfs*23
82ORG
*
+/M
UNC119
M: c.7delG p.V3*
51ORG
+/M1
*
M1/M2
M1/M2
+/M2
USH2A
M1: c.1724G>A p.C575Y
M2: c.2276GT p.C759F
73ORG
+/M1
+/M2
*
M1/M2
M1/M2
USH2A
M1: c.2276GT p.C759F
M2: c.13010C>T p.T4337M
